# Contrasting foliar nitrogen nutrition of coexisting temperate and boreal trees across a modest temperature cline

**DOI:** 10.1016/j.fmre.2024.10.004

**Published:** 2024-10-18

**Authors:** Yang Tang, Enzai Du, Josep Peñuelas, Peter B. Reich

**Affiliations:** a State Key Laboratory of Earth Surface Processes and Resource Ecology, Faculty of Geographical Science, Beijing Normal University, Beijing 100875, China; b School of Natural Resources, Faculty of Geographical Science, Beijing Normal University, Beijing 100875, China; c Consejo Superior de Investigaciones Científicas, Global Ecology Unit CREAF-CSIC-UAB, 08913 Cerdanyola del Vallès, Catalonia, Spain; d Centre de Recerca Ecològica i d'Aplicacions Forestals, 08913 Cerdanyola del Vallès, Catalonia, Spain; e Institute for Global Change Biology, University of Michigan, Ann Arbor, MI 48109, USA; f Department of Forest Resources, University of Minnesota, St. Paul, MN 55108, USA

**Keywords:** Nitrogen availability, Nitrogen isotope, Temperate-boreal forest ecotone, Temperature gradient, Mongolian oak, Dahurian larch

## Abstract

The temperate-boreal forest ecotone represents a transition zone from temperate to boreal forest where nitrogen (N) is frequently limiting tree growth. However, the spatial patterns and potential drivers of plant N nutrition and soil N availability remain poorly understood. To address this, we conducted a field investigation along a temperate-boreal forest ecotone in northeastern China, characterized by a modest mean annual temperature gradient (∼1 °C) within the range of current climate warming. Our goal was to evaluate the spatial variation in foliar N nutrition and soil N availability, and the potential driving factors for Mongolian oak (*Quercus mongolica*) and Dahurian larch (*Larix gmelinii*), the dominant trees of the local temperate and adjacent boreal forests, respectively. Our results revealed no significant spatial trend in topsoil N availability across the sampling transect. Foliar N concentration was significantly higher, but foliar δ^15^N was lower, for Mongolian oak than Dahurian larch. Foliar N concentration for Mongolian oak increased significantly toward the boreal forest, driven by lower mean annual temperature and mean annual precipitation, with no significant trend observed for Dahurian larch. Moreover, foliar Δδ^15^N (foliar δ^15^N−soil δ^15^N) decreased significantly for Mongolian oak as it approached the boreal forest, while it increased significantly for Dahurian larch toward the temperate forest. Notably, foliar N concentration, δ^15^N, and Δδ^15^N for Dahurian larch increased significantly with an increasing basal area proportion of Mongolian oak. Our findings demonstrate contrasting patterns of foliar N nutrition between co-occurring temperate and boreal trees across a temperate-boreal forest ecotone with a modest climatic gradient. These results underscore the importance of incorporating interspecific interactions to enhance our understanding of future N cycling in southern boreal forests in the context of climate warming.

## Graphical abstract

**Figure:**
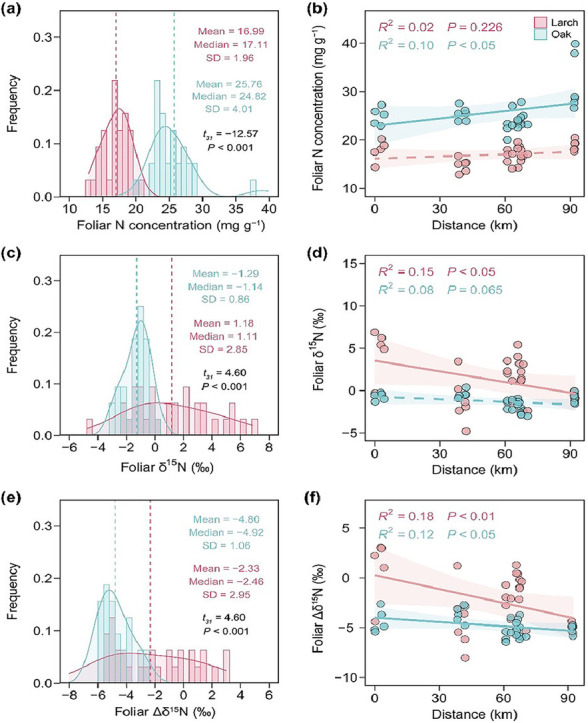

Based on field investigations conducted in a temperate-boreal forest ecotone in northeastern China, we evaluated the patterns and potential drivers of soil nitrogen (N) availability and foliar N nutrition for Mongolian oak (Quercus mongolica) and the co-occurring boreal Dahurian larch (Larix gmelinii). Our study revealed contrasting foliar N nutrition between these temperate and boreal tree species across a modest climatic gradient and suggested that the presence of migrating temperate trees can positively affect the foliar N nutrition of adjacent boreal conifers under climate warming. These findings enhance our understanding of spatial patterns in soil N availability and foliar N nutrition within temperate-boreal forest ecotones and highlight the importance of interspecific interactions in predicting future N cycling dynamics in southern boreal forests amid ongoing climate change.Frequency distribution and spatial variation of foliar N concentration (a, b), δ^15^N (c, d) and Δδ^15^N (e, f) for Mongolian oak and Dahurian larch across the temperate-boreal forest ecotone. Distance represents the distance from each sampling plot to the start point of the sampling transect. SD, standard deviation. Δδ^15^N represents the difference between foliar δ^15^N and soil δ^15^N.

## 1 Introduction

The temperate-boreal forest ecotone, a transition zone between temperate and boreal forest biomes, is particularly sensitive to climate change [[1], [2], [3]]. Due to climatic warming, temperate broadleaved trees have been migrating beyond their current northernmost or uppermost boundaries [4,5], leading to shifts in species composition across the temperate-boreal forest ecotone [5,6]. These shifts in the mix of temperate broadleaved trees and boreal coniferous trees can theoretically alter nutrient cycling, as interspecific differences in nutrient use and litter chemistry can affect plant nutrition and soil nutrients availability over time [7,8]. Nitrogen (N), an essential nutrient, significantly limits tree growth in both temperate and boreal forests [9,10]. The changes in species composition driven by climate warming, along with their subsequent effects on soil N availability, have been documented in various high-altitude and high-latitude ecotones, such as alpine treelines and boreal forest-tundra ecotones [[11], [12], [13]]. However, the variation in soil N availability and foliar N nutrition across the temperate-boreal forest ecotone, and the potential impacts of the increasing dominance of temperate trees on foliar N nutrition for co-occurring boreal trees, remain less well understood. This knowledge gap hinders our ability to accurately predict future changes in N cycling and vegetation productivity in southern boreal forests under climate warming.

The availability of soil N in boreal forests is strongly limited by cold climates due to lowered rates of microbial mineralization and other N transformation processes (e.g., nitrification) [14,15]. Soil N availability is theoretically expected to increase from colder boreal forest toward warmer temperate forest with accelerated mineralization of soil N. Additionally, the increasing proportion of temperate broadleaved trees from boreal forest to temperate forest may further accelerate the mineralization of soil N by producing more decomposable litter (e.g., higher N concentration and lower concentrations of recalcitrant compounds), and thus enhance the expected increase in N availability across temperature gradients [8], although not all evidence supports these hypotheses [16]. The spatial variation in the availability of soil N across the temperate-boreal forest ecotone can be mediated by other vegetational, topographic and soil conditions [17]. For example, N pools of soil and plant N gradually accumulate with stand age and thus affect soil N availability over time [18,19]. Similarly, differences in litter productivity (driven by climate, species characteristics, or both) can influence annual N inputs into soils and hence drive soil N availability [20,21]. Spatial variations in the slope and aspect of a forest stand may also affect soil N availability by modifying the microclimate and the capacity to retain soil nutrients [22,23]. The hypothesized spatial gradients of soil N availability and the roles of the aforementioned drivers, however, have rarely been tested across temperate-boreal forest ecotones.

The foliar N nutrition for co-occurring temperate and boreal trees may change across the temperate-boreal forest ecotone in acclimation to, and perhaps adaptation to, the spatial gradients of the aforementioned soil N availability and additional abiotic and biotic factors, and such variation can occur within as little as a 1−2 °C temperature gradient [24]. A warmer climate in combination with higher water availability not only improves soil N availability but also increases the demand of N for tree growth, thus jointly affecting the foliar N balance across the climatic gradients [[25], [26], [27]]. An increasing proportion of broadleaved trees may increase the availability of soil N for co-occurring conifers, whereas the broadleaved trees and co-occurring conifers also compete intensively for N in N-limited ecosystems and potentially decrease N availability and foliar N nutrition for neighboring conifers [28]. Furthermore, N use strategies can also contribute to determine the level of foliar N nutrition for both temperate broadleaved trees and conifers [24]. For example, mycorrhizal fungal associations can affect plant N nutrition since the host plants partially depend on mycorrhizal fungi for N acquisition especially at low N availabilities [14,29,30]. Temperate trees may also adjust their allocation and use of N toward colder climates from temperate forest to boreal forest, such as allocating more N to foliage to increase metabolic activity and growth rate [31,32]. In contrast, boreal trees may show a different trend across the temperate-boreal forest ecotone, likely having lower foliar N concentrations in colder regions as part of the whole-plant strategy of having longer needle longevity in response to both cold and slower growth rates [33]. The hypothesized interspecific differences and biogeographical variations in the foliar N nutrition of temperate and boreal trees are complex and require further investigation especially within a temperature gradient similar as the range of global climate warming (e.g., < 2 °C).

N isotopic signatures (i.e. δ^15^N) of plants and soil have been widely used to characterize N cycle in terrestrial ecosystems, and to indicate its response to changing N availability [30,34,35]. The availability of soil N for terrestrial plants has been found to strongly correlate with δ^15^N in soil and foliage [30]. Increasing supply of soil N in natural ecosystems generally accelerates N cycling (e.g., mineralization, nitrification, denitrification and N losses) and enriches the heavier ^15^N in the soil substrate and plant foliage due to the fractionation of N isotopes [29]. Increased dominance of temperate broadleaved trees with N-rich litters may increase soil δ^15^N by accelerating N cycling, and thereby increase foliar δ^15^N for co-occurring boreal trees. Moreover, as soil N availability increases, plants tend to be less dependent on mycorrhizal fungi, which preferentially transfers ^15^N-depleted N to host plants, partly resulting in a decrease in foliar δ^15^N [36]. Foliar δ^15^N for co-occurring temperate broadleaved trees and boreal conifers may also have different spatial trends across the temperate-boreal forest ecotone due to their distinct capacities for N acquisition and utilization.

The northern Greater Khingan Mountains in northeastern China cover the southernmost part of the Eurasian boreal forest [37]. Mongolian oak (*Quercus mongolica*), a broadleaved tree of the northern temperate forest in this region, is expanding toward the boreal forest, which is dominated by Dahurian larch (*Larix gmelinii*), due to rapid climatic warming [4,38,39]. The gradients of co-occurring Mongolian oak and Dahurian larch across the temperate-boreal forest ecotone thus provide a unique research platform to address the aforementioned questions. Based on a field investigation across a modest temperature gradient (∼1 °C) within the temperate-boreal forest ecotone on the eastern slope of the Greater Khingan Mountains, we explored the spatial patterns and their potential driving factors of soil N availability and foliar N nutrition of temperate Mongolian oak and co-occurring boreal Dahurian larch. The aims of this work were therefore to characterize the spatial variation in soil N availability and foliar N nutrition for occurring Mongolian oak and Dahurian larch across the temperate-boreal forest ecotone, and further uncovered the potential driving factors, including climatic, edaphic and vegetational conditions. Specifically, we tested the following hypotheses: (*i*) the availability of soil N increases from the boreal forest toward the temperate forest and this pattern is dominated by the temperature gradient across the temperate-boreal forest ecotone; (*ii*) foliar N nutrition for co-occurring Mongolian oak and Dahurian larch, surrogated by foliar N concentration, δ^15^N and Δδ^15^N (foliar δ^15^N−soil δ^15^N), have different spatial trends across the temperate-boreal forest ecotone due to their distinct capacities of N use; and (*iii*) increased abundance of Mongolian oak can improve the foliar N nutrition for co-occurring Dahurian larch by increasing the availability of soil N.

## 2 Materials and methods

### 2.1 Study area and sampling transect

**Fig. 1:**
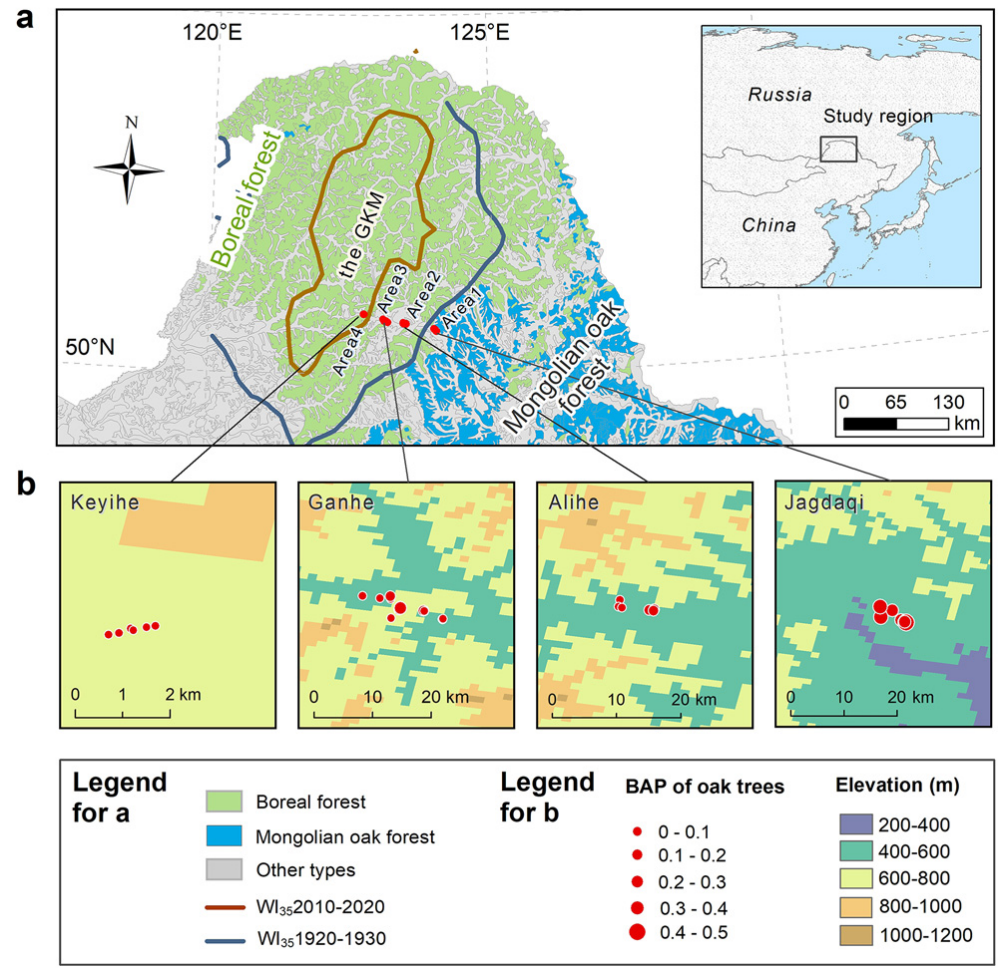
**Study area and sampling transect across the temperate-boreal forest ecotone in northeastern China (a, b).** The sampling transect, vegetation distribution and the isoline of the threshold warm index (i.e. 35 °C month, calculated as the sum of monthly mean temperatures > 5 °C) for the reproduction and regeneration of Mongolian oak (see Tang et al. [4] for more details) (a). The sampling sites and the topography of the study region (b). The isoline of the threshold warm index has moved toward the boreal forest from the 1920s (WI_35_ 1920−1930, blue) to the 2010s (WI_35_ 2010−2020, brown) (a), and Mongolian oak has rapidly migrated into boreal forest [4]. The size of the red dots in panel (b) indicates the basal-area proportion (BAP) for Mongolian oak in each sampling plot. GKM, the Greater Khingan Mountains. Map approval number: GS(2023)2762.

This study was conducted across a temperate-boreal forest ecotone on the eastern slope of northern Greater Khingan Mountains (latitude 50°10′–53°27′ N and longitude 119°36′–126°37′ E), which represents a part of the southernmost edge of Eurasian boreal forest (Fig. 1a). The boreal forest in this region is dominated by Dahurian larch (*L. gmelinii*) [40]. Mongolian oak (*Q. mongolica*), a dominant broadleaved tree in the adjacent temperate forest, has been migrating rapidly toward the boreal forest during the last century due to substantial climatic warming [4]. Dahurian larch and Mongolian oak are both associated with ectomycorrhizal fungi [41], but Mongolian oak has a more advanced root system than does Dahurian larch [42].

We established a sampling transect (latitude 50°28′–50°40′ N and longitude 122°42′–123°59′E) across the temperate-boreal forest ecotone, almost perpendicular to the isolines of the threshold warmth index required for the reproduction of Mongolian oak (see Fig. 1a and Table S1 for more details). The sampling transect was 90 km long (Fig. 1). Mean annual temperature (MAT) ranged from −1.75 to −0.65 °C and mean annual precipitation (MAP) ranged from 480 to 550 mm across the sampling transect (Table S1; Fig. S1). The altitude (above sea level) increased from 470 m in the east to 650 m in the west. The sizes and ages of the co-occurring Mongolian oak and Dahurian larch had opposite trends along the sampling transect (Fig. S2). Specifically, Mongolian oak decreased significantly in age (measured at ∼5 cm height above the ground, decreasing from 85 ± 8 yrs in Area 1 to 20 ± 2 yrs in Area 4), and basal area (at a height of 1.3 m, ranging from 0.02 ± 0.009 m^2^ to 0.3 ± 0.1 m^2^) toward the southern boreal forest (Fig. S2). In contrast, the cambial age (measured at breast height) and basal area (at a height of 1.3 m) of the Dahurian larch decreased significantly toward the temperate forest (Fig. S2). The larch forest grows on brown coniferous forest soil [43], with the depth of the mineral soil layer varying from 20 to 40 cm. Soil pH ranges between 5.0 and 6.5, with no significant spatial trend along the sampling transect (Fig. S1). The ambient N deposition is low at ∼5 kg ha^−1^ yr^−1^ [44].

### 2.2 Field sampling and laboratory measurements

In the summer of 2020, we conducted an investigation across 32 forest plots (20 m × 20 m each) in four sampling areas—Jagdaqi, Alihe, Ganhe, and Keyihe (hereafter referred to as Areas 1 to 4) (Fig. 1; Table S1). The sampling transect began in Area 1 at the plot closest to pure Mongolian oak forests. We selected 5 to 15 representative plots in each area where Mongolian oak and Dahurian larch co-occurred. The forest plots in each area were chosen based on the following criteria: (*i*) Dahurian larch, the dominant species, co-occurs with Mongolian oak populations; (*ii*) the plots are situated on low to moderate slopes (ranging from 0° to 20°); and (*iii*) the plots are free from recent disturbances (e.g., fire damage and logging) and are located > 100 m away from the forest edge. All the selected plots were included in subsequent analyses, differing from a previous study that assessed the migration rate of Mongolian oak using five sampling forest plots containing the oldest Mongolian oak trees in each area [4]. For each plot, we recorded geographical information (latitude, longitude, elevation, and slope) and the coverage of understory plants (herbaceous plants and shrubs). We measured the diameter at breast height (DBH) for all individuals taller than 1.3 m using a diameter tape. In each plot, we collected foliar samples from the six largest, healthy Mongolian oak and Dahurian larch trees, sampling from the upper sunward crown using an averruncator. A total of 384 foliar samples were obtained across the transect—192 for each species. To determine the age of Dahurian larch trees, we sampled tree-ring cores from the eight largest individuals per plot at breast height using an increment borer. For Mongolian oak, we sampled stem discs from the three largest and three relatively smaller trees at a height of 5 cm above the ground. Additionally, five topsoil samples (0−10 cm depth) were randomly collected from each plot using a soil auger. Foliar and soil samples were separately mixed to create a composite sample per plot for subsequent laboratory analysis.

Foliar samples were oven-dried at 65 °C for 48 h to a constant weight, then milled using a mixer ball mill (20 Hz for 5 min) (MM400; Retsch, Haan, Germany) and sieved through a 100-mesh sieve. Topsoil samples were air-dried at room temperature and passed through a 2-mm sieve to remove root fragments, coarse debris, and gravel. Fine roots were manually removed, and the topsoil samples were milled using an agate mortar grinder (RM200; Retsch, Haan, Germany) before being passed through a 100-mesh sieve. Topsoil pH was measured in aqueous suspensions (water: soil = 2.5:1, volume/weight) of the air-dried samples using a pH meter (pHS-25; INESA, Shanghai, China). Total carbon (C) and nitrogen (N) concentrations and abundances of ^15^N (δ^15^N) of the milled foliar and topsoil samples were measured using an elemental analyzer (Elemental Analysis System GmbH; Elementar, Hanau, Germany) coupled with a stable isotope ratio mass spectrometer (Delta V; Thermo Fisher, Massachusetts, USA). Topsoil C:N ratios were then calculated to indicate the topsoil N availability [45,46]. The analytical errors (i.e. standard deviations) of the isotope measurements were evaluated based on the values for replicated measurements of reference material. The analytical errors were 0.25‰ for δ^15^N and 0.13‰ for total N concentration, respectively, implying a good consistency and accuracy of measurements. The abundance of ^15^N (δ^15^N) was calculated as Eq. 1,

(1) $$\delta^{15}N=\left[\frac{R_{sample}}{R_{standard}}-1\right]\times \;1000\%$$

where *R_sample_* is the ^15^N/^14^N ratio in the sample, and *R_standard_* is the ^15^N/^14^N ratio in atmospheric N_2_.

To control the potentially different background topsoil δ^15^N signatures among sampling forest plots [30,47], we calculated the difference in δ^15^N between foliage and topsoil (i.e. Δδ^15^N) according to Eq. 2,

(2) $$\Delta \delta^{15}N=\delta^{15}N_{foliage}-\delta^{15}N_{soil}$$

where δ^15^N_foliage_ represents ^15^N abundance in foliar samples, and δ^15^N_soil_ represents ^15^N abundance in topsoil samples.

Tree-ring counts for Dahurian larch and Mongolian oak were measured using a LINTAB 5.0 system (RINNTECH, Heidelberg, Germany). The dating of tree-rings was performed and corrected using COFECHA software [48]. The average tree-ring counts for the eight larch trees in each plot were used to estimate stand age. All laboratory analyses were conducted at the Analysis and Test Center, State Key Laboratory of Earth Surface Processes and Resource Ecology, Beijing Normal University.

### 2.3 Data on potential drivers

To explore the potential drivers of the varying availability of soil N and foliar N nutrition for co-occurring Mongolian oak and Dahurian larch, we compiled data on nine explanatory variables: (*i*) two climatic variables, MAT and MAP; (*ii*) two topographical variables, slope and aspect; (*iii*) one soil variable, pH; and (*iv*) four vegetational variables, understory plant coverage (Under_cov), stand age, total basal area of each plot (Plot BA), and the basal-area proportion (BAP) for Mongolian oak. Basal-area proportion in each sampling plot was calculated according to Eq. 3,

(3) $$\text{BAP}=\frac{BA_{oak}}{BA_{oak}+BA_{larch}}\times \;100\%$$

where BA_oak_ and BA_larch_ are the total basal areas for Mongolian oak and Dahurian larch in each plot, respectively.

Meteorological stations were rare in our sampling areas and there was only one within Area 1 (i.e. Jagdaqi). Data on MAT and MAP (1980−2018) for each sampling plot were thus derived from CHELSA v2.1 at a resolution of 30 arc-seconds [49]. We also derived observed data on MAT and MAP (1980−2018) for Area 1 from the nearby meteorological station (China Meteorological Data Service Center, http://data.cma.cn) and conducted a statistical comparison with the CHELSA dataset. Both MAT and MAP for Area 1 correlated strongly between these two datasets (MAT: *R^2^* = 0.94, *P* < 0.001; MAP: *R^2^* = 0.79, *P* < 0.001; Fig. S3), implying a reliability for CHELSA dataset in the study area. Other explanatory variables were measured during the field sampling.

### 2.4 Statistical analysis

A Shapiro-Wilk test was conducted to test normality of total N, δ^15^N and Δδ^15^N in foliar and soil samples, and all these variables were found to follow a normal distribution (Table S2). The interspecific differences in foliar N concentration, δ^15^N, and Δδ^15^N between Mongolian oak and co-occurring Dahurian larch were tested using a paired *t*-test. Linear regression analyses were conducted to assess the spatial changes in the availability of topsoil N (i.e. soil C:N ratio and δ^15^N), tree foliar N nutrition (i.e. foliar N concentration, δ^15^N, and Δδ^15^N), and their potential driving factors along the sampling transect (i.e. with the distance from the starting plot Area 1–1).

We performed a model-selection analysis using Akaike's information criteria corrected for small samples to evaluate the importance of potential drivers for the availability of topsoil N (i.e. soil C:N ratio and δ^15^N) and tree foliar N nutrition (i.e. foliar N concentration, δ^15^N, and Δδ^15^N) [50] . All nine potential drivers were used in the analyses due to the low collinearity amongst them (absolute *Pearson's r* < 0.7; Fig. S4). The relative importance of each driver was estimated as the sum of the Akaike weights for models in which the drivers were included, and a cut-off of 0.8 was used to identify the important drivers [50]. The variance inflation factor (VIF) was used to indicate the multicollinearity for the driving factors in the best models (VIF < 3 suggests no statistical collinearity) [51]. A conditional regression analysis was used to demonstrate the role of the important drivers while keeping the other drivers fixed [52]. The contribution of each driving factor was calculated by averaging the variance over the orderings of the regressors [53]. All statistical analyses and visualizations were performed using R sofware (version 4.2.3, R Development Core Team, 2023) with a significance level of *P* < 0.05. Data are shown as means ± standard deviations unless otherwise noted.

## 3 Results

### 3.1 Variations in the availability of soil N across the sampling transect

**Fig. 2:**
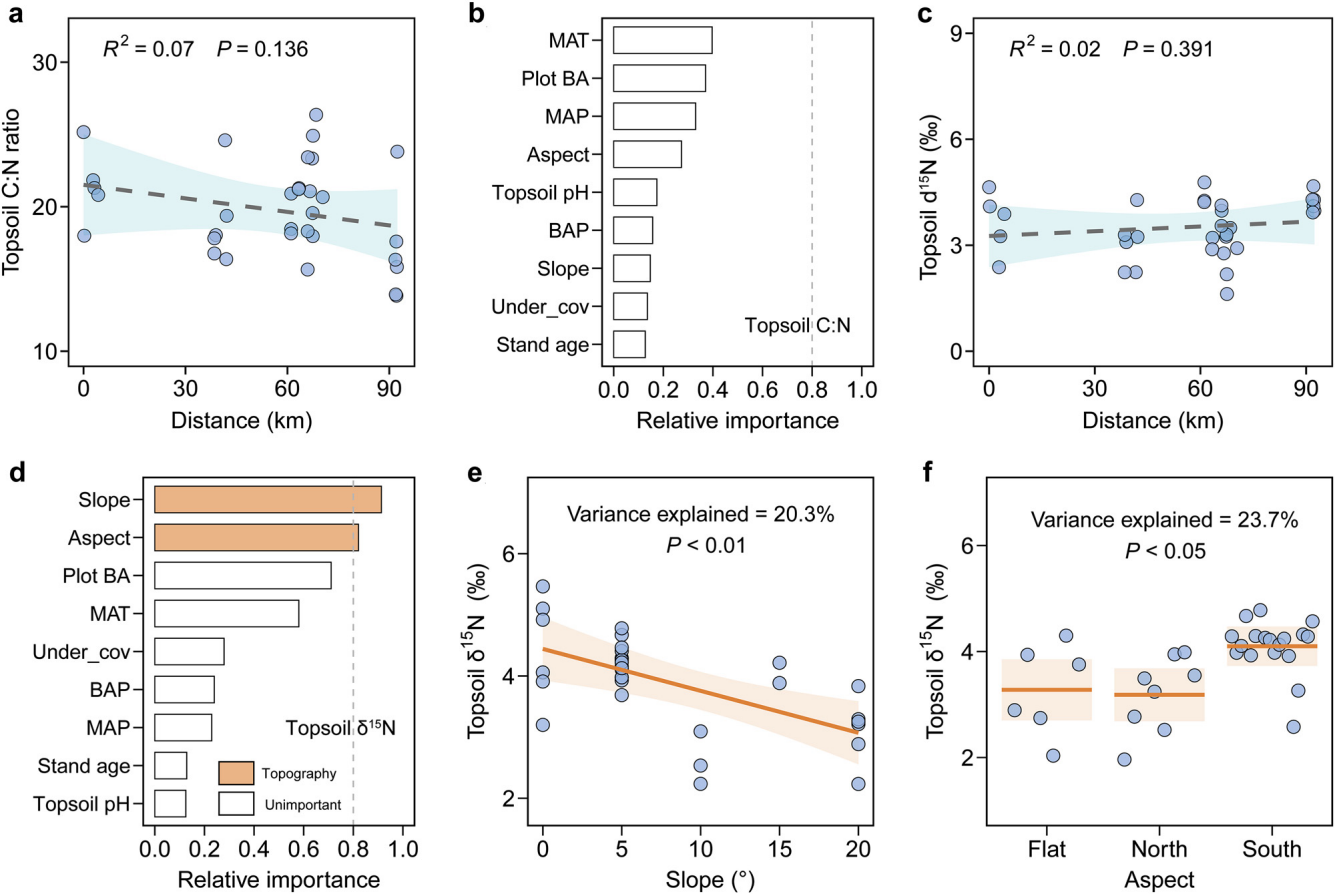
**Spatial variations in the topsoil C:N ratio and δ^15^N (a, c), relative importance of potential predictors (b, d) and conditional regression plots for the important drivers (e, f).** The shading in (a), (c), (e) and (f) represents the 95% confidence interval of the model fit. See Table S3 for a summary of the models. Abbreviations: MAT, mean annual temperature; MAP, mean annual precipitation; Plot BA, total basal area of the sampling plot; Under_cov, understory plant coverage; BAP, the basal-area proportion for Mongolian oak; Aspect, the aspect of a sampling plot, i.e. north-facing, south-facing or flat.

The topsoil C:N ratio ranged from 13.8 to 26.4 (19.5 ± 3.3; Fig. S5) but had no significant trend across the sampling transect (*P* = 0.14; Fig. 2a). Surprisingly, the spatial variations in the topsoil C:N ratio was not significantly explained by the nine potential drivers (Fig. 2b; Table S3). Topsoil δ^15^N (ranging from 1.6‰ to 5.7‰; mean = 3.5‰ ± 0.8‰; Fig. S5) also had no significant trend across the sampling transect (*P* = 0.39; Fig. 2c), and its spatial variation was mainly attributable to the varied slope and aspect of the sampling plots (explaining 38% of the total variance) (Fig. 2d; Table S3). Specifically, conditional regression analyses found that topsoil δ^15^N decreased significantly with the slope of the sampling plot (explaining 20% of the variance, *P* < 0.01; Fig. 2e). Topsoil δ^15^N also varied significantly with the aspect of the sampling plot (explaining 24% of the variance, *P* < 0.05; Fig. 2f), with the highest values occurring on south-facing aspects.

### 3.2 Interspecific differences in foliar nitrogen nutrition across the transect

**Fig. 3:**
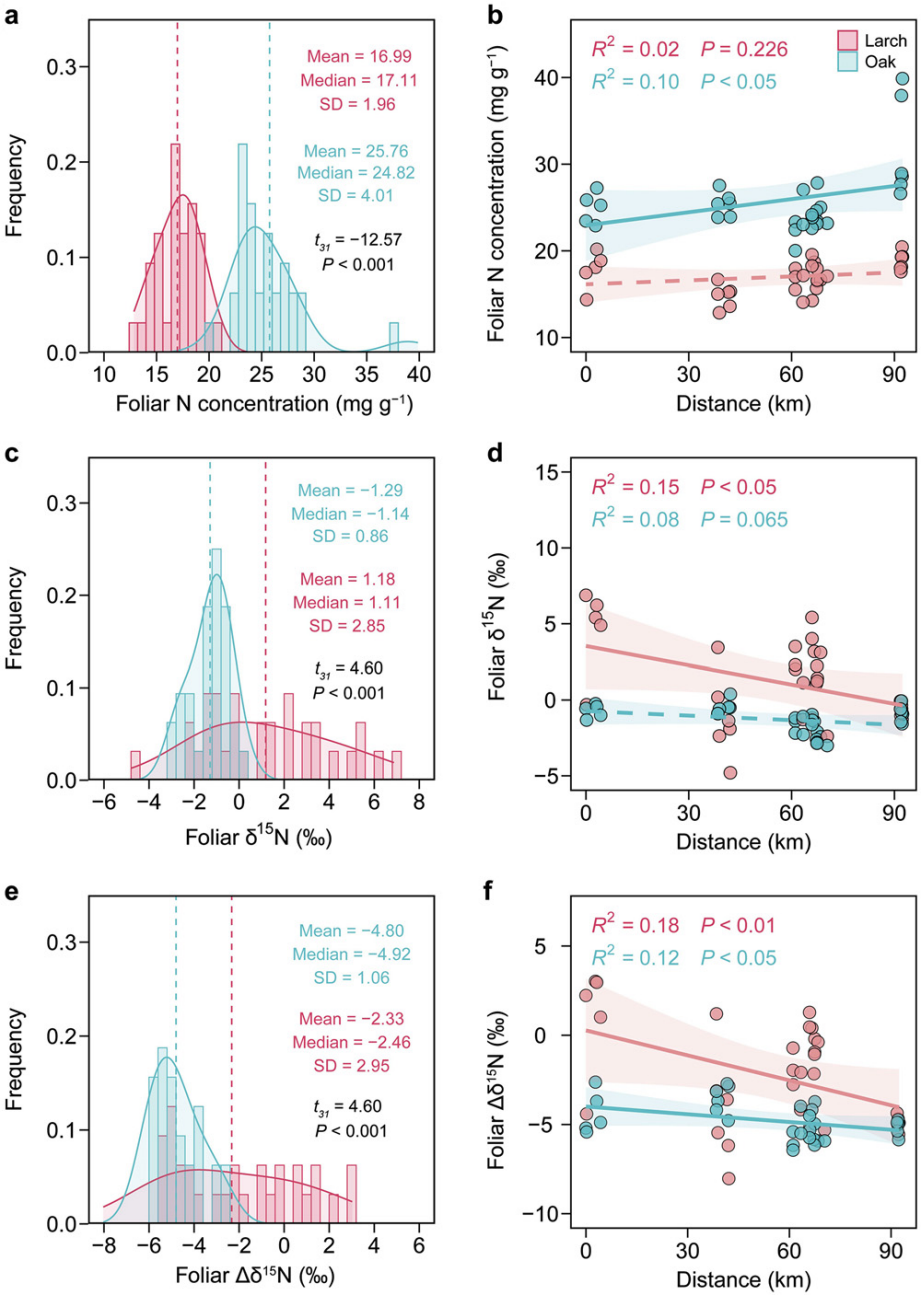
**Frequency distribution and spatial variation of foliar N concentration (a, b), δ^15^N (c, d), and Δδ^15^N (e, f) for Mongolian oak and Dahurian larch across the temperate-boreal forest ecotone.** A paired *t*-test was used to determine the interspecific difference. Distance represents the distance from each sampling plot to the start point of the sampling transect (i.e. Area 1–1, see Fig. 1). SD, standard deviation.

Foliar N concentration of Dahurian larch (17.0 ± 2.0 mg *g*^−1^) was significantly lower than the co-occurring Mongolian oak (25.8 ± 4.0 mg *g*^−1^) (paired *t*-test, *df* = 31, *t* = −12.57, *P* < 0.001; Fig. 3a). The foliar C:N ratio of Dahurian larch (30.8 ± 6.4) was accordingly higher than Mongolian oak (18.4 ± 2.2) (*df* = 31, *t* = 10.88, *P* < 0.001; Fig. S6). The foliar N concentration for Mongolian oak increased significantly toward the boreal forest (*P* < 0.05; Fig. 3b), but the foliar N concentration for Dahurian larch showed no significant trend across the sampling transect (*P* = 0.23). Foliar δ^15^N was significantly higher for Dahurian larch (1.2‰ ± 2.9‰) than Mongolian oak (−1.3‰ ± 0.9‰) (*df* = 31, *t* = 4.60, *P* < 0.001; Fig. 3c). Foliar δ^15^N of Dahurian larch increased significantly toward the temperate forest (*P* < 0.05) but it had marginally significant trend for Mongolian oak across the sampling transect (*P* = 0.07; Fig. 3d). Moreover, foliar Δδ^15^N for Mongolian oak (−4.8‰ ± 1.1‰) was significantly lower than for Dahurian larch (−2.3‰ ± 3.0‰) (*df* =31, *t* = 4. 60, *P* < 0.001; Fig. 3e). Foliar Δδ^15^N for Mongolian oak decreased significantly toward the boreal forest (*P* < 0.05; Fig. 3f), and increased significantly for Dahurian larch toward the temperate forest (*P* < 0.01; Fig. 3f).

### 3.3 Drivers of variations in foliar N concentration across the transect

**Fig. 4:**
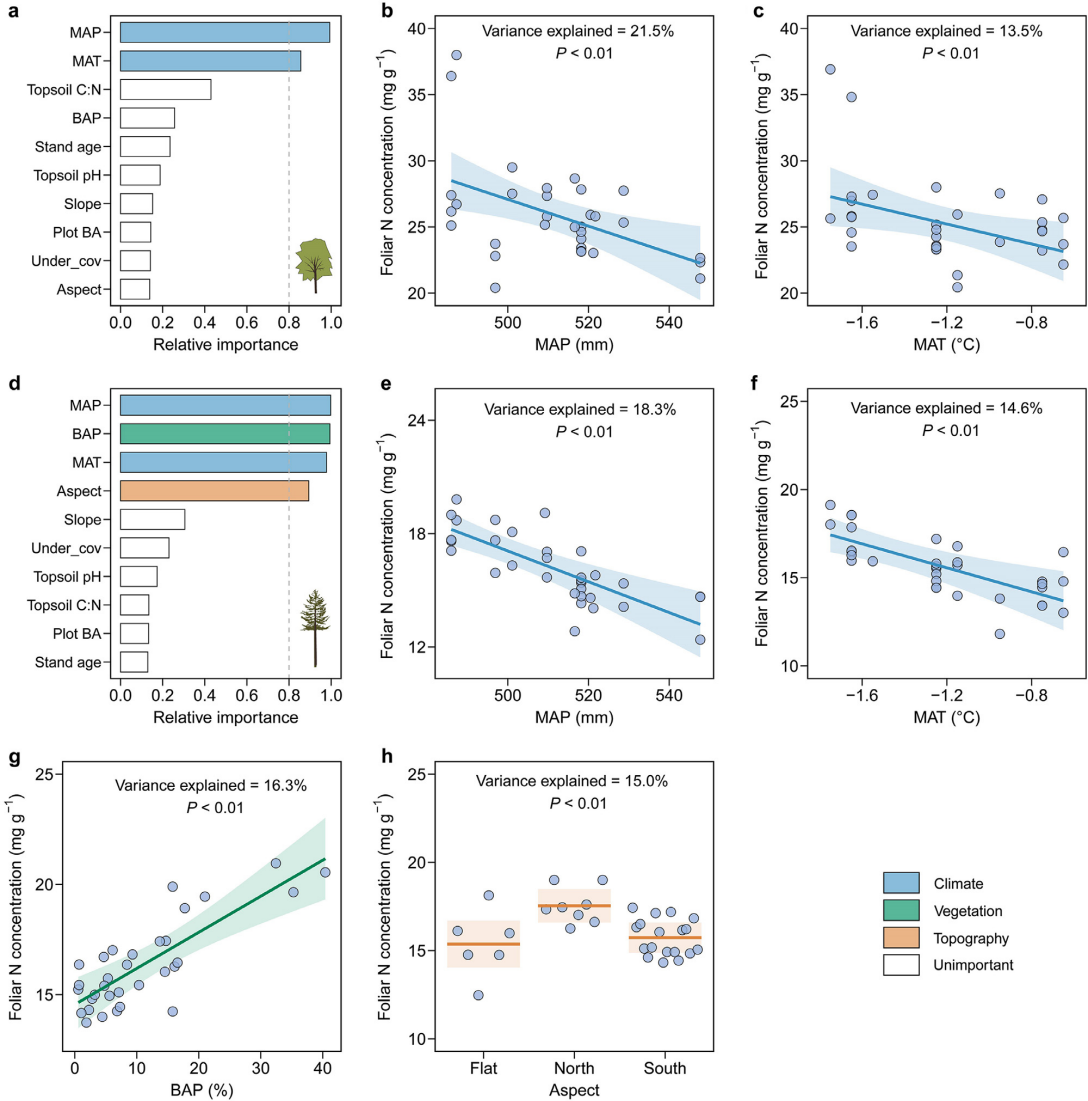
**Relative importance of potential drivers of foliar nitrogen concentration of Mongolian oak and Dahurian larch (a, d) and conditional regression plots for the important drivers.** The shading in (*b*−*c*) and (*e*−*h*) represents the 95% confidence interval of the model fit. See Table S4 for a summary of the model. See the caption for Fig. 2 for abbreviations.

The spatial trend in the foliar N concentration for Mongolian oak was mainly regulated by MAP and MAT (explaining 35% of the total variance; Fig. 4a), which had weak collinearity (*Pearson's r* = 0.09; Fig. S4). Specifically, the foliar N concentration for Mongolian oak increased significantly as MAP decreased (explaining 22% of the variance, *P* < 0.01; Fig. 4b) and MAT decreased (explaining 14% of the variance, *P* < 0.01; Fig. 4c). In addition to the climatic factors, the spatial variation of the foliar N concentration for Dahurian larch was also explained by the basal-area proportion of the co-occurring Mongolian oak and the aspect of the sampling plot (explaining 57% of the total variance; Fig. 4d). A conditional regression analysis showed that the foliar N concentration of Dahurian larch increased significantly with lower MAP (explaining 18% of the variance, *P* < 0.01; Fig. 4e), lower MAT (explaining 15% of the variance, *P* < 0.01; Fig. 4f) and higher basal-area proportion of the co-occurring Mongolian oak (explaining 16% of the variance, *P* < 0.01; Fig. 4g). The foliar N concentration for Dahurian larch also varied significantly with the aspect of the sampling plot (explaining 15% of the variance, *P* < 0.01), with higher values occurring on north-facing aspects (Fig. 4h).

### 3.4 Drivers of variations in foliar δ^15^N across the transect

**Fig. 5:**
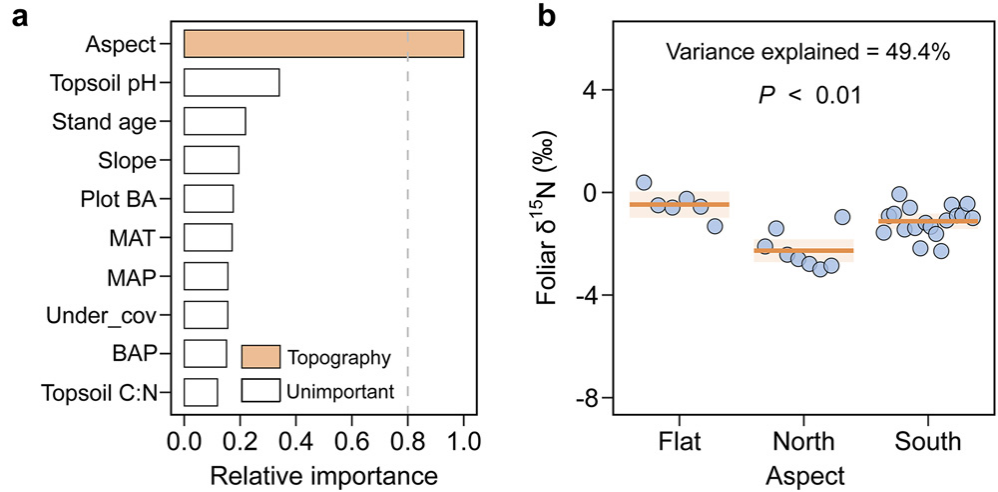
**Relative importance of potential drivers of foliar δ^15^N for Mongolian oak (a), and regression plot showing the change of foliar δ^15^N with stand aspect (b).** The shading indicates the 95% confidence interval of the linear model fit. See Table S5 for a summary of the model. See the caption for Fig. 2 for abbreviations.

**Fig. 6:**
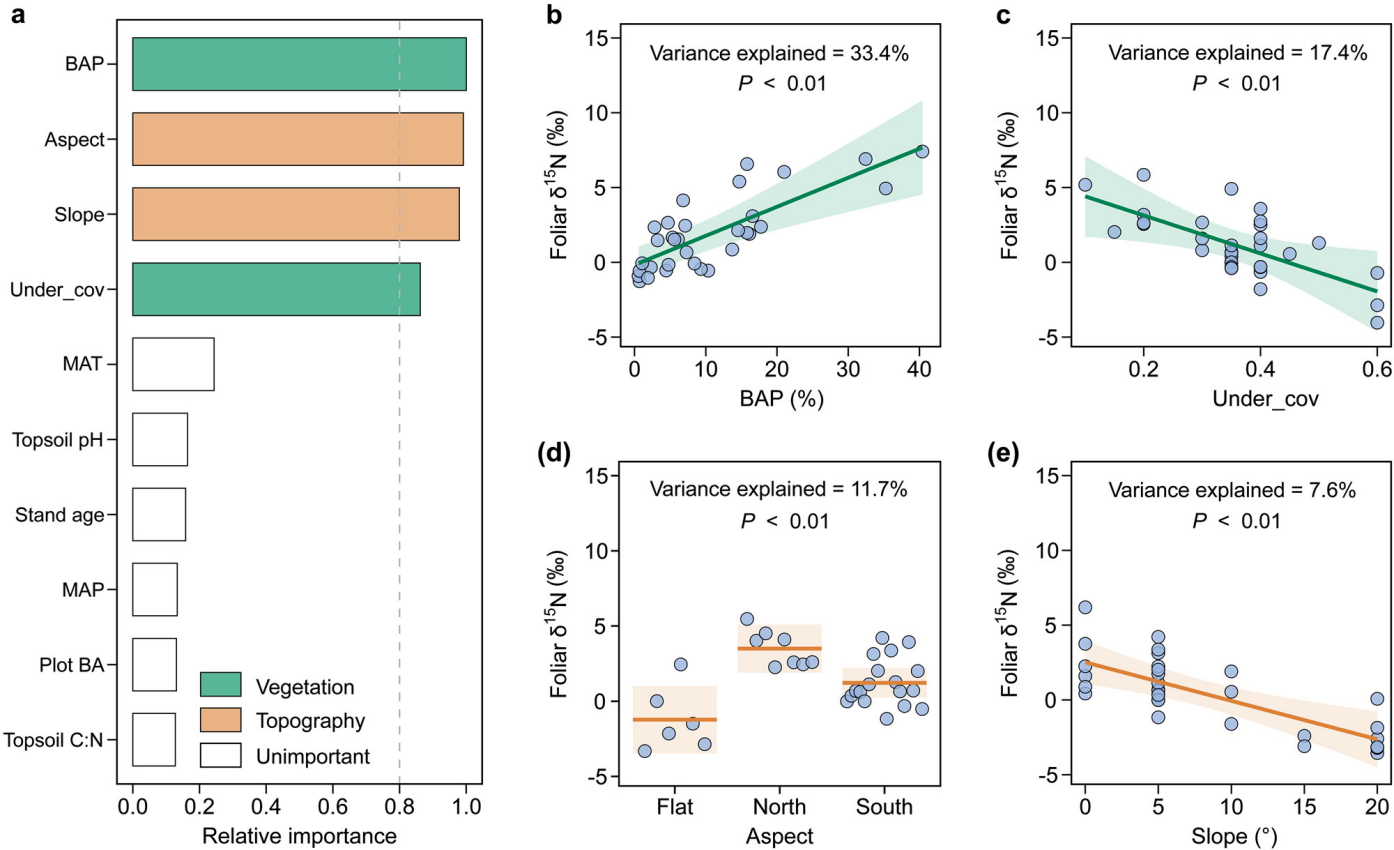
**Relative importance of potential drivers of foliar δ^15^N for Dahurian larch (a), and conditional regression plots for the basal-area proportion (BAP) of Mongolian oak (b), understory plant coverage (c), stand aspect (d) and slope (e).** The shading indicates the 95% confidence interval of the linear model fit. See Table S5 for a summary of the model. See the caption for Fig. 2 for abbreviations.

The spatial variation of foliar δ^15^N for Mongolian oak was mainly explained by the aspect of the sampling plots (explaining 49 % of the variance, *P* < 0.01; Fig. 5a), with the lowest values occurring on north-facing aspects (Fig. 5b). In contrast, the spatial trend of foliar δ^15^N for Dahurian larch was jointly explained by the basal-area proportion for Mongolian oak, the coverage of understory plants and the aspect and slope of the sampling plots (explaining 65% of the total variance; Fig. 6a). Specifically, foliar δ^15^N for Dahurian larch increased significantly with higher basal-area proportion of the co-occurring Mongolian oak (explaining 33% of the variance, *P* < 0.01; Fig. 6b), lower coverage of understory plants (explaining 17% of the variance, *P* < 0.01; Fig. 6c) and higher slope (explaining 8% of the variance, *P* < 0.01; Fig. 6e). Foliar δ^15^N for Dahurian larch also varied significantly with aspect (explaining 12% of the variance, *P* < 0.01), with higher values occurring on north-facing aspects (Fig. 6d).

### 3.5 Drivers of variations in foliar Δδ^15^N across the transect

**Fig. 7:**
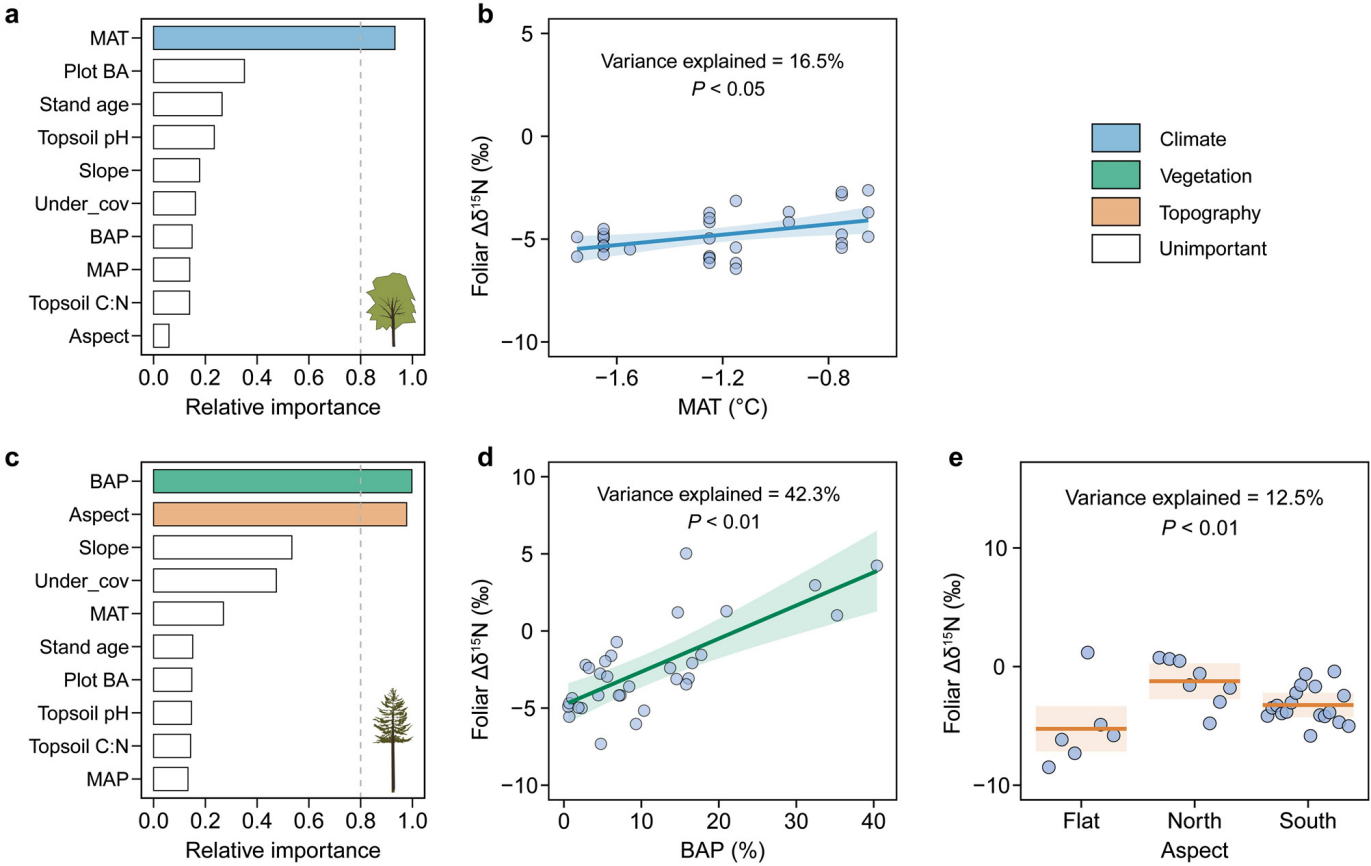
**Relative importance of potential drivers of foliar Δδ^15^N (a, c) of Mongolian oak and Dahurian larch and conditional regression plots with the important drivers.** The shading represents the 95% confidence interval of the model fit. See Table S6 for a summary of the model. See the caption for Fig. 2 for abbreviations.

The spatial trend of foliar Δδ^15^N for Mongolian oak was exclusively explained by MAT (explaining 17% of the variance; Fig. 7b). Specifically, foliar Δδ^15^N for Mongolian oak increased significantly with higher MAT (*P* < 0.05; Fig. 7b). In contrast, the spatial variation in foliar Δδ^15^N for Dahurian larch was mainly explained by the basal-area proportion of Mongolian oak and stand aspect (explaining 55% of the total variance; Fig. 7c). Conditional regression analyses further revealed a significant increase in foliar Δδ^15^N for Dahurian larch with higher basal-area proportion of Mongolian oak (explaining 42% of the variance, *P* < 0.01; Fig. 7d). Additionally, foliar Δδ^15^N for Dahurian larch varied significantly with stand aspect (explaining 13% of the variance, *P* < 0.01), with higher values occurring on north-facing aspects (Fig. 7e).

## 4 Discussion

### 4.1 Nonsignificant gradient of topsoil N availability across the temperate-boreal forest ecotone

In contrast to our first hypothesis, we found that the availability of N in topsoil, surrogated by the C:N ratio and δ^15^ N, had no significant trend across the transect of temperate-boreal forest ecotone. The slopes and aspects of the sampling plots accounted for a considerable proportion (38%) of the spatial variation in soil δ^15^N (Fig. 2), implying an important role of topographical conditions in determining the spatial variation in the soil N availability within this ecotone. The nonsignificant gradient of the availability of topsoil N was likely due to three causes. First, the climatic conditions especially MAT varied within a relatively narrow range across the sampling transect (∼1 °C; Fig. S1) and may not have caused a major pattern of soil N cycling and hence detectable changes in N availability. Second, N is limiting in the study area, so a minor increase in available N (e.g., inorganic N) under higher temperature and/or increased abundance of Mongolian oak can be readily assimilated by plants and therefore there was a negligibly detectable increase in these two surrogates of soil N availability. Third, topographical conditions exert a strong heterogeneity in the cycling of topsoil N by regulating microclimates and hydrologic processes [22,54]. Specifically, lower stand slopes on south-facing aspects, corresponding to higher temperatures and better retention of topsoil N, may be favorable to soil N mineralization and hence its higher availability to trees [23,55]. Thus, the effect of spatial variation in topographical conditions may potentially mask the role of modest climatic gradients across the temperate-boreal forest ecotone.

### 4.2 Interspecific differences of foliar N concentrations across the temperate-boreal forest ecotone

Our results indicated that Mongolian oak and co-occurring Dahurian larch exhibited distinct foliar N-nutrition signatures, represented by N concentration and δ^15^N, across the temperate-boreal forest ecotone (Fig. 3). These distinct N-nutrition signatures suggest differences in foliar structure and capacities for N acquisition and utilization between the broadleaved Mongolian oak and coniferous Dahurian larch [41,42]. These differences may also be attributable to other species-specific traits. For example, Mongolian oak possesses a more efficient fine-root system with a higher specific root length and root density compared to Dahurian larch [56]. Despite similar ectomycorrhizal associations, Mongolian oak has an advantage over Dahurian larch in acquiring soil N [[56], [57], [58]]. In addition to interspecific differences in root traits, which are closely linked to tree N absorption, N allocation strategies also contribute to the variation in foliar N nutrition concentrations [31,59]. Specifically, temperate oaks growing in cold climates likely allocate more N to foliage to support biochemical processes such as photosynthesis [60].

We also observed distinct spatial patterns of foliar N concentrations for Mongolian oak and co-occurring Dahurian larch across the temperate-boreal forest ecotone, partially supporting our second hypothesis. Specifically, the foliar N concentration for Mongolian oak increased significantly toward the boreal forest, whereas Dahurian larch showed no detectable trend in foliar N concentrations across the ecotone. This pattern of increasing foliar N concentration for Mongolian oak from warmer to colder climates aligns with some previous studies [60,61], but not all [24]. Generally, plants growing in colder temperatures may exhibit higher leaf N concentrations as they allocate more N-rich compounds to leaves to support metabolic processes (like photosynthesis and respiration), aiding their acclimation to colder climates [31,59]. However, colder soils and lower soil N availability in colder habitats may lead to lower foliar N concentrations [24]. Moreover, these responses may be non-linear with respect to temperature across large geographic scales, varying among species and functional groups [31], indicating that the power of different mechanisms influencing foliar N status may differ from extreme cold to cool to warm climate conditions.

In contrast to Mongolian oak, Dahurian larch trees across the ecotone are at their southern distributional limit [40]. Their growth in this region is primarily constrained by water availability rather than by cold environments [62]. The higher MAP toward the temperate forest may benefit the growth of Dahurian larch, potentially diluting foliar N nutrition and leading to a lower foliar N concentration [63]. Additionally, a higher MAT can increase plant transpiration rates, subsequently reducing the capacity for N absorption and upward transportation, thereby decreasing the foliar N concentration [64,65]. Our results also indicated that a higher proportion of Mongolian oak significantly improved foliar N nutrition for co-occurring Dahurian larch, evidenced by the higher foliar N concentration (Fig. 4), partially supporting our third hypothesis. This beneficial effect was likely due to the higher litter quality (e.g., lower C:N ratio) of Mongolian oak, which accelerates litter decomposition and N mineralization [66,67]. These findings also demonstrate the predominance of the positive effect of Mongolian oak on foliar N nutrition of co-occurring Dahurian larch, outweighing its negative impact due to competition stress with increasing basal area proportion (Fig. 4g). However, the benefits from Mongolian oak were also likely offset in part by the impacts of MAP and MAT, as discussed above, resulting in a nonsignificant trend in foliar N concentration for Dahurian larch across the ecotone.

### 4.3 Interspecific differences of foliar ^15^N abundance and Δδ^15^N across the temperate-boreal forest ecotone

Mongolian oak and Dahurian larch had distinct patterns of foliar δ^15^N and Δδ^15^N across the ecotone (Figs. 3d, f), consistent with our second hypothesis. Specifically, foliar δ^15^N for Mongolian oak did not have significant trends along the transect, but foliar δ^15^N for Dahurian larch increased toward the temperate forest. The pattern of foliar δ^15^N for Mongolian oak is exclusively regulated by the local aspect of the sampling plot, i.e. the warmer microclimate on flat plots or south-facing aspects benefits the release of N and hence the higher availability of N to the trees [23,55]. Furthermore, the foliar Δδ^15^N for Mongolian oak was found to significantly decrease with the decrease in MAT (Fig. 7b), which aligns with the previously observed pattern of decreasing foliar Δδ^15^N with decreasing MAT [29]. The depletion in foliar ^15^N for temperate Mongolian oak may partially be attributable to the stronger association with ectomycorrhizal fungi to improve N acquisition toward the colder boreal forest [14].

The pattern of increasing foliar δ^15^N for Dahurian larch with the increasing proportion of Mongolian oak clearly suggests a beneficial effect of Mongolian oak on foliar N nutrition for Dahurian larch, which partially supports our third hypothesis. This pattern may be due to the aforementioned mechanism that the increasing abundance of Mongolian oak improves the litter chemistry and hence accelerates the rates of N mineralization and the losses of topsoil ^14^N, thereby increasing δ^15^N of the soil and larch foliage. Such positive effects of broadleaved tree species on foliar N nutrition for resident trees have also been observed in other ecotones and forest stands of mixed tree species [[68], [69], [70]]. Additionally, the increase in foliar Δδ^15^N for Dahurian larch, accompanied by an increasing proportion of neighboring Mongolian oak, provides evidence for enhanced uptake of ^15^N-enriched soil inorganic N sources for Dahurian larch under more interspecific competition (represented by basal area proportion) from Mongolian oak, which is in accordance with previous results focused on grassland communities [34,71]. Moreover, the pattern of increasing foliar Δδ^15^N for Dahurian larch with an increasing abundance of Mongolian oak may also reveal a less fractionation against ^15^N during the N uptake for Dahurian larch (Fig. 7d). The possible causes likely include a decrease in the reliance of Dahurian larch on mycorrhizal fungi for N uptake due to the beneficial effects of the increasing dominance of Mongolian oak on the foliar N nutrition for Dahurian larch, indicated by positive correlation between foliar N and δ^15^N for Dahurian larch and dominance of Mongolian oak (Fig. 4, Fig. 6), and thereby a more enriched foliar ^15^N. Our results also imply a negative effect of the understory plants and the slope of the sampling plot on foliar δ^15^N for Dahurian larch across the ecotone (Fig. 6), i.e. the increasing abundance of understory plants tends to intensify the competition for topsoil N nutrients with larch trees [72,73], and increased slopes of sampling plots generally causes more erosive losses of topsoil N [22,23,47], both of which can decrease the N availability for the Dahurian larch.

### 4.4 Implications and outlook for further research

Climate warming not only can directly accelerate soil N mineralization by stimulating soil microbial activity [74], but also promotes the expansion of temperate trees, which improve litter quality [4]. Both factors can contribute to increased soil N availability and consequently, enhanced foliar N nutrition for boreal trees. However, climatic warming may also exacerbate moisture and temperature stress in southern boreal forests [62,75]. A recent tree-ring analysis has revealed widespread decline in the growth of Dahurian larch in the southern Asian boreal forest due to moisture stress [76], similar to observed responses elsewhere in southern boreal forests [77,78]. Thus, an important question remains: will the positive effects of temperate Mongolian oak on N nutrition accelerate the growth of Dahurian larch, particularly in the context of moisture stress at this temperate-boreal forest ecotone? Additionally, rapid climate warming may reduce foliar N nutrition for temperate trees by limiting N uptake due to moisture stress-related reductions in N diffusion and flow in soils [79,80], which could lead to decreased N release from the litter of temperate trees. These findings suggest complex impacts of climate warming, along with associated shifts in species composition, on N cycling, foliar N nutrition, and tree growth in southern boreal forests. Future manipulative experiments, such as the removal or introduction of Mongolian oak across temperature gradients, are necessary to provide deeper insights into the mechanisms driving changes in N cycling, foliar N nutrition, and boreal forests growth in response to the expansion of temperate Mongolian oak under climate warming. From a forest management perspective, the assisted introduction of oaks under current climate warming conditions appears plausible as a strategy to improve N nutrition for boreal larch.

Several additional uncertainties remain in our analysis. For instance, global gridded climate datasets (e.g., CHELSA) may not accurately capture the micrometeorological conditions of forest stands [49], potentially obscuring the effects of climatic conditions on the spatial variation in soil N availability across a modest temperature gradient. Moreover, we did not measure rooting depths, which could influence N nutrition for Mongolian oak and Dahurian larch. Rooting depth may impact foliar N isotopic signatures since the δ^15^N of soil N sources tends to increase along the vertical soil profile [30,81]. The difference in rooting depth between Mongolian oak and Dahurian larch may therefore partially explain the differences in foliar N isotopic signatures. Additionally, different forms of N in the soil are generally considered to exhibit distinct N isotope signatures [71]. Specifically, nitrate typically has higher δ^15^N compared to ammonia and organic N, due to more fractionation against ^15^N during denitrification [82]. Consequently, investigating the N sources in the soils (e.g., nitrogen, ammonia, and dissolved organic N) and the N uptake patterns of Mongolian oak and co-occurring Dahurian larch across the ecotone is necessary to better understand the spatial trends and driving factors for soil and foliar N isotope signatures.

The potential driving factors considered in our analysis were not strongly colinear (absolute *Pearson's r* < 0.7; Fig. S4), but a modest correlation between the basal-area proportion of Mongolian oak and MAT (*Pearson's r* = 0.53) still suggests the potential effects of temperature on the dominance of Mongolian oak across this ecotone. Specifically, Mongolian oak is likely to establish earlier and grow faster in warmer boreal forest stands, thus having more potential to accelerate N cycling. Warmer climates inherently directly accelerate litter decomposition and N mineralization [83,84], leading to an increase in foliar N nutrition for Dahurian larch. Therefore, further manipulative experiments are necessary to separate the biological effects of migrating Mongolian oak from the direct effects of climate.

## 5 Conclusion

Based on a field investigation of a temperate-boreal forest ecotone in northeastern China, we evaluated the patterns and potential drivers of soil N availability and foliar N nutrition for Mongolian oak and co-occurring boreal Dahurian larch. We found that the availability of topsoil N had no detectable trend across the ecotone with a modest climatic gradient. Foliar N concentrations were higher but δ^15^N was lower for Mongolian oak than co-occurring Dahurian larch. The foliar N concentration for Mongolian oak increased toward the boreal forest, likely due to the physiological acclimation of N investment in the cold environment [60,61]. In contrast, Dahurian larch did not have a significant trend in foliar N concentration across the ecotone. Foliar ^15^N signatures for Mongolian oak and Dahurian larch also showed distinct patterns across the ecotone. We further discovered that the increased dominance of Mongolian oak had a beneficial effect on the status of foliar N for Dahurian larch. These findings improve our understanding of the spatial patterns of soil N availability and foliar N nutrition for trees across the temperate-boreal forest ecotone and highlight the effects of changing species composition on N nutrition for trees across the temperate-boreal forest ecotone.

## Appendix Supplementary materials

Image, application 1
