# Supplementary material for: Contrasting foliar nitrogen nutrition of coexisting temperate and boreal trees across a modest temperature cline
Source: Fundam Res. 2024 Oct 18;6(2):929–39. doi: 10.1016/j.fmre.2024.10.004 (PMC13069620; doi:10.1016/j.fmre.2024.10.004)

*Supplementary information*

**Contrasting foliar nitrogen nutrition of coexisting temperate and boreal trees across a modest temperature cline**

Yang Tang^1, 2^, Enzai Du^1, 2*^, Josep Peñuelas^3, 4^, Peter B. Reich^5, 6^

^1^State Key Laboratory of Earth Surface Processes and Resource Ecology, Faculty of Geographical Science, Beijing Normal University, Beijing 100875, China

^2^School of Natural Resources, Faculty of Geographical Science, Beijing Normal University, Beijing 100875, China

^3^CSIC, Global Ecology Unit CREAF-CSIC-UAB, 08913 Cerdanyola del Vallès, Catalonia, Spain

^4^CREAF. 08913 Cerdanyola del Vallès, Catalonia, Spain

^5^Institute for Global Change Biology, University of Michigan, Ann Arbor, MI, USA

^6^Department of Forest Resources, University of Minnesota, St. Paul, MN, USA

*Corresponding author: Enzai Du; Tel: +8610-58808085; Email: [enzaidu@bnu.edu.cn](mailto:enzaidu@bnu.edu.cn)

This file contains **Tables S1−S6 & Figures S1−S6.**

**Table S1. A summary of soil, climate, topographical and vegetation conditions in 32 sampling plots in this study.** Abbreviations: MAP, mean annual precipitation; MAT: mean annual temperature; BAP1: basal area proportion of Mongolian oak in each plot; BAP2: basal area proportion of other co-occurring trees except for Mongolian oak and Dahurian larch (e.g., *Betula platyphylla*) in each plot.

| **Area** | **Plot ID** | **Longitude** | **Latitude** | **Altitude** | **Aspect** | **MAT** | **MAP** | **Slope** | **BAP1 (%)** | **BAP2** | **Soil pH** |
| --- | --- | --- | --- | --- | --- | --- | --- | --- | --- | --- | --- |
|  |  | **(°E)** | **(°N)** | **(m)** |  | **(℃)** | **(mm)** | **(°)** |  | **(%)** |  |
| Area 1 | Area 1-1 | 123.963 | 50.497 | 472 | Flat | -0.65 | 509.2 | 0 | 15.8 | 0.9 | 5.47 |
|  | Area 1-2 | 123.986 | 50.477 | 475 | Flat | -0.75 | 528.7 | 0 | 40.4 | 2.3 | 5.24 |
|  | Area 1-3 | 123.984 | 50.478 | 479 | Flat | -0.75 | 528.7 | 0 | 13.7 | 1.2 | 5.08 |
|  | Area 1-4 | 123.944 | 50.486 | 481 | South | -0.65 | 501.1 | 20 | 32.4 | 1.8 | 5.88 |
|  | Area 1-5 | 123.942 | 50.504 | 470 | South | -0.65 | 501.1 | 15 | 35.3 | 2.6 | 6.07 |
| Area 2 | Area 2-1 | 123.410 | 50.575 | 490 | Flat | -0.75 | 509.7 | 0 | 9.3 | 0.1 | 5.29 |
|  | Area 2-2 | 123.409 | 50.566 | 497 | Flat | -0.75 | 509.7 | 0 | 7.1 | 0.2 | 5.24 |
|  | Area 2-3 | 123.413 | 50.564 | 511 | South | -0.75 | 509.7 | 10 | 8.4 | 0 | 5.68 |
|  | Area 2-4 | 123.451 | 50.561 | 471 | Flat | -0.95 | 516.6 | 0 | 15.8 | 0 | 5.47 |
|  | Area 2-5 | 123.458 | 50.562 | 500 | South | -1.15 | 521.7 | 20 | 14.7 | 0.1 | 5.62 |
|  | Area 2-6 | 123.458 | 50.560 | 493 | South | -0.95 | 516.6 | 20 | 14.5 | 0.6 | 5.36 |
| Area 3 | Area 3-1 | 123.056 | 50.616 | 564 | North | -1.25 | 518.2 | 10 | 2.8 | 0.2 | 5.18 |
|  | Area 3-2 | 123.057 | 50.580 | 607 | North | -1.25 | 518.2 | 5 | 6.1 | 0 | 5.54 |
|  | Area 3-3 | 123.040 | 50.611 | 580 | North | -1.25 | 518.2 | 5 | 3.2 | 0 | 5.49 |
|  | Area 3-4 | 123.057 | 50.610 | 579 | North | -1.25 | 518.2 | 5 | 4.4 | 0 | 5.25 |
|  | Area 3-5 | 123.136 | 50.579 | 545 | South | -1.15 | 496.9 | 5 | 4.7 | 0.7 | 5.58 |
|  | Area 3-6 | 123.136 | 50.578 | 550 | South | -1.15 | 496.9 | 5 | 5.3 | 0 | 5.6 |
|  | Area 3-7 | 123.137 | 50.579 | 567 | South | -1.15 | 496.9 | 5 | 6.8 | 0.5 | 5.41 |
|  | Area 3-8 | 123.056 | 50.616 | 587 | North | -1.25 | 518.2 | 10 | 5.6 | 0 | 5.27 |
|  | Area 3-9 | 123.013 | 50.615 | 588 | North | -1.25 | 518.2 | 20 | 4.8 | 0 | 5.62 |
|  | Area 3-10 | 123.056 | 50.614 | 595 | North | -1.25 | 518.2 | 15 | 16.1 | 0 | 5.45 |
|  | Area 3-11 | 123.072 | 50.598 | 580 | North | -1.65 | 547.7 | 5 | 17.7 | 0.6 | 5.61 |
|  | Area 3-12 | 123.070 | 50.593 | 656 | South | -1.65 | 547.7 | 5 | 10.4 | 0 | 5.46 |
|  | Area 3-13 | 123.072 | 50.596 | 641 | South | -1.65 | 547.7 | 5 | 21.0 | 0 | 5.52 |
|  | Area 3-14 | 123.107 | 50.592 | 587 | South | -1.55 | 520.5 | 20 | 16.6 | 0 | 5.65 |
|  | Area 3-15 | 123.108 | 50.591 | 578 | South | -1.25 | 521.2 | 20 | 7.3 | 0 | 5.82 |
| Area 4 | Area 4-1 | 122.714 | 50.668 | 649 | South | -1.75 | 487.3 | 5 | 0.7 | 0 | 5.72 |
|  | Area 4-2 | 122.713 | 50.667 | 646 | South | -1.75 | 487.3 | 5 | 0.6 | 0 | 5.33 |
|  | Area 4-3 | 122.717 | 50.668 | 637 | South | -1.65 | 486 | 5 | 0.7 | 0.1 | 5.61 |
|  | Area 4-4 | 122.717 | 50.668 | 632 | South | -1.65 | 486 | 5 | 2.3 | 0 | 5.7 |
|  | Area 4-5 | 122.720 | 50.669 | 648 | South | -1.65 | 486 | 5 | 1.9 | 0 | 5.74 |
|  | Area 4-6 | 122.721 | 50.669 | 654 | South | -1.65 | 486 | 5 | 1.1 | 0 | 5.49 |

**Table S2. A summary of Shapiro-Wilk test for variables associated with topsoil N availability and foliar N nutrition.**

| **Variables** | ***W*-value** | ***P*-value** |
| --- | --- | --- |
| **Topsoil** |  |  |
| C:N ratio | 0.971 | 0.527 |
| δ^15^N | 0.952 | 0.162 |
| **Mongolian oak** |  |  |
| N concentration | 0.932 | 0.053 |
| Foliar δ^15^N | 0.959 | 0.252 |
| Foliar Δδ^15^N | 0.944 | 0.098 |
| **Dahurian larch** |  |  |
| N concentration | 0.977 | 0.702 |
| Foliar δ^15^N | 0.972 | 0.544 |
| Foliar Δδ^15^N | 0.956 | 0.210 |

**Table S3. A summary of the models for topsoil C:N ratio and δ^15^N.** Abbreviations: VIF, the variance inflation factor. VIFs < 3 for all predictors suggest no strong multicollinearity among them.

| **Parameter** | **VIF** | **Estimates** | **Std. Error** | ***t*-value** | ***P*-value** | **Variance explained (%)** |
| --- | --- | --- | --- | --- | --- | --- |
|  |  |  |  |  |  |  |
| **Topsoil C:N** |  |  |  |  |  |  |
| **-** | **-** | - | - | - | - | - |
| **Topsoil δ^15^N** |  |  |  |  |  |  |
| Intercept |  | 3.620 | 0.263 | 9.635 |  |  |
| Slope | 1.209 | -0.069 | 0.020 | -3.409 | < 0.01 | 20.3 |
| South | 1.099 | 0.822 | 0.364 | 2.260 | < 0.05 | 23.7 |

**Table S4. A summary of the models for foliar N content.** Abbreviations: MAT, mean annual temperature; MAP, mean annual precipitation; Under_cov, understory plant coverage; BAP, the basal area proportion of Mongolian oak in each sampling plot; VIF, the variance inflation factor. VIFs < 3 for all predictors suggest no strong multicollinearity among them.

| **Drivers** | **VIF** | **Estimates** | **Std. Error** | ***t*-value** | ***P*-value** | **Variance explained (%)** |  |
| --- | --- | --- | --- | --- | --- | --- | --- |
|  |  |  |  |  |  |  |  |
| **Mongolian oak** |  |  |  |  |  |  |  |
| Intercept |  | 4.846 | 0.614 | 7.894 |  |  |  |
| MAP | 1.009 | -0.003 | 0.001 | -2.914 | < 0.01 | 21.5 |  |
| MAT | 1.009 | -0.125 | 0.057 | -2.193 | < 0.05 | 13.5 |  |
| **Dahurian larch** |  |  |  |  |  |  |  |
| Intercept |  | 52.033 | 8.274 | 6.289 |  |  |  |
| MAP | 1.300 | -0.081 | 0.017 | -4.849 | < 0.05 | 18.3 |  |
| North | 1.214 | 2.170 | 0.812 | 2.671 | < 0.05 | 15.0 |  |
| MAT | 1.462 | -3.398 | 0.912 | -3.726 | < 0.001 | 14.6 |  |
| BAP | 1.407 | 16.368 | 0.032 | 5.111 | < 0.001 | 16.3 |  |

**Table S5. A summary of the models for foliar δ^15^N.** Abbreviations: MAT, mean annual temperature; BAP, the basal area proportion of Mongolian oak in each sampling plot; Under_cov, understory plant coverage; VIF, the variance inflation factor. VIFs < 3 for all predictors suggest no strong multicollinearity among them.

| **Drivers** | **VIF** | **Estimates** | **Std. Error** | ***t*-value** | ***P*-value** | **Variance explained (%)** |  |
| --- | --- | --- | --- | --- | --- | --- | --- |
|  |  |  |  |  |  |  |  |
| **Mongolian oak** |  |  |  |  |  |  |  |
| Intercept |  | -0.472 | 0.251 | -1.884 |  |  |  |
| North | - | -1.795 | 0.332 | -5.413 | < 0.001 | 49.4 |  |
| South | - | -0.652 | 0.289 | -2.254 | < 0.05 |  |  |
| **Dahurian larch** |  |  |  |  |  |  |  |
| Intercept |  | 3.111 | 2.834 | 1.098 |  |  |  |
| BAP | 1.427 | 0.194 | 0.043 | 4.512 | < 0.001 | 33.4 |  |
| North | 1.331 | 4.729 | 1.214 | 3.896 | < 0.001 | 11.7 |  |
| Slope | 1.546 | -0.258 | 0.068 | -3.798 | < 0.001 | 7.6 |  |
| Under_cov | 1.903 | -12.715 | 4.862 | -2.615 | < 0.05 | 17.4 |  |

**Table S6. A summary of the models for foliar Δδ^15^N.** Abbreviations: MAT, mean annual temperature; BAP, the basal area proportion of Mongolian oak in each sampling plot; VIF, the variance inflation factor. VIFs < 3 for all predictors suggest no strong multicollinearity among them.

| **Drivers** | **VIF** | **Estimates** | **Std. Error** | ***t*-value** | ***P*-value** | **Variance explained (%)** |
| --- | --- | --- | --- | --- | --- | --- |
| **Mongolian oak** |  |  |  |  |  |  |
| Intercept |  | -3.274 | 0.5973 | -5.481 |  |  |
| MAT | - | 1.259 | 0.4719 | 2.669 | < 0.05 | 16.5 |
| **Dahurian larch** |  |  |  |  |  |  |
| Intercept |  | -6.794 | 1.08 | -6.29 |  |  |
| BAP | 1.112 | 0.215 | 0.04 | 5.496 | < 0.001 | 42.3 |
| North | 1.112 | 4.013 | 1.184 | 3.388 | < 0.01 | 12.5 |

**Figure S1**. **Spatial variation of potential drivers across the sampling transect**. Distance represents the geographical distance from each sampling plots to the start point of the sampling transect (i.e., Area 1-1). Tree ages of Dahurian larch and Mongolian oak were estimated as the counts of tree rings at breast height (e.g., 1.4 meters) and at a standard height of ~5 cm above ground, respectively. Abbreviations: MAT, mean annual temperature; MAP, mean annual precipitation; Plot BA, total basal area of the sampling plot; Under_cov, understory plant coverage; BAP, the basal area proportion of Mongolian oak. The shades represent the 95% confidence intervals of the model fit.


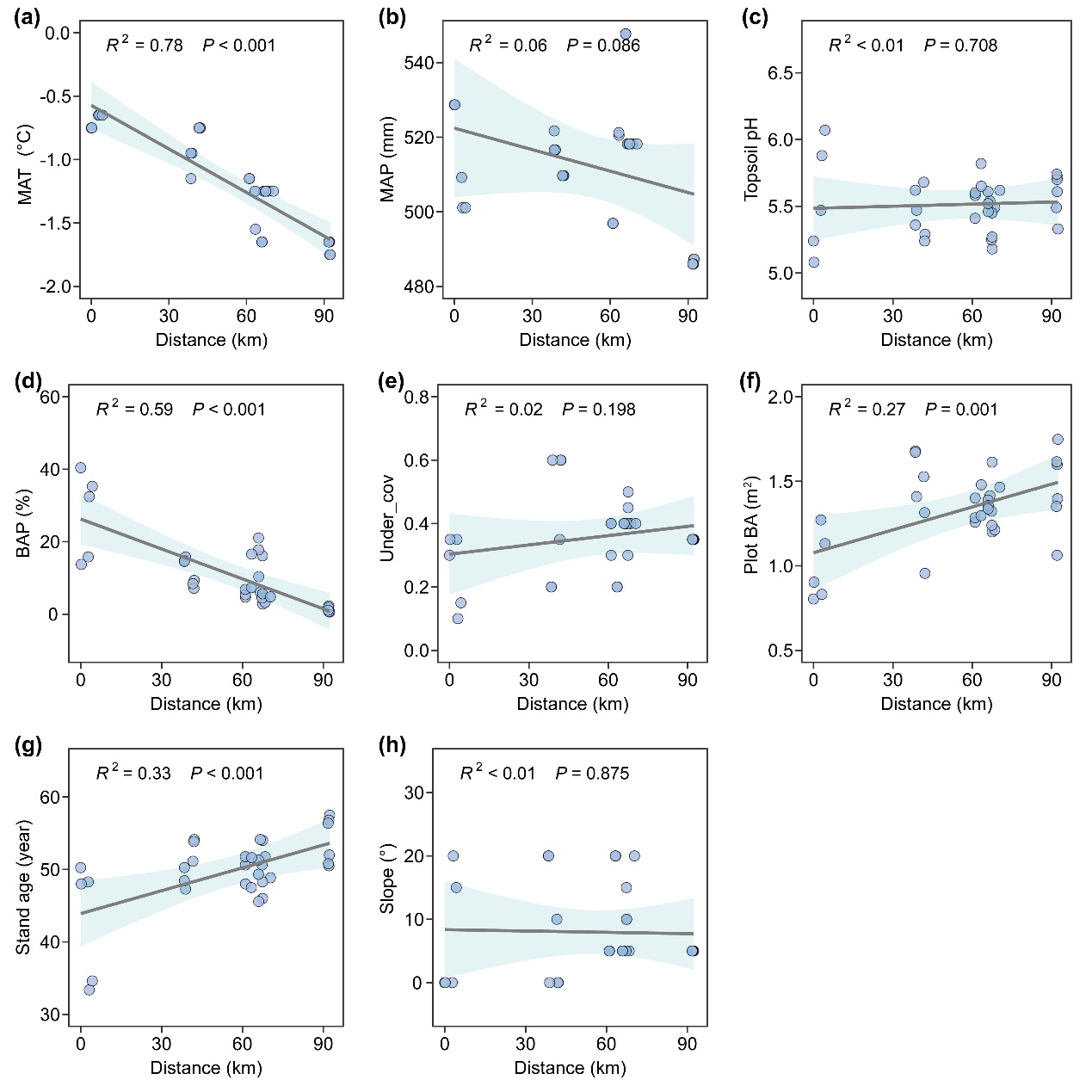


**Figure S2**. **Spatial variation of tree age, DBH and basal area across the sampling transect**. The shades represent the 95% confidence intervals of the model fit. See the caption of Figure S1 for abbreviations.


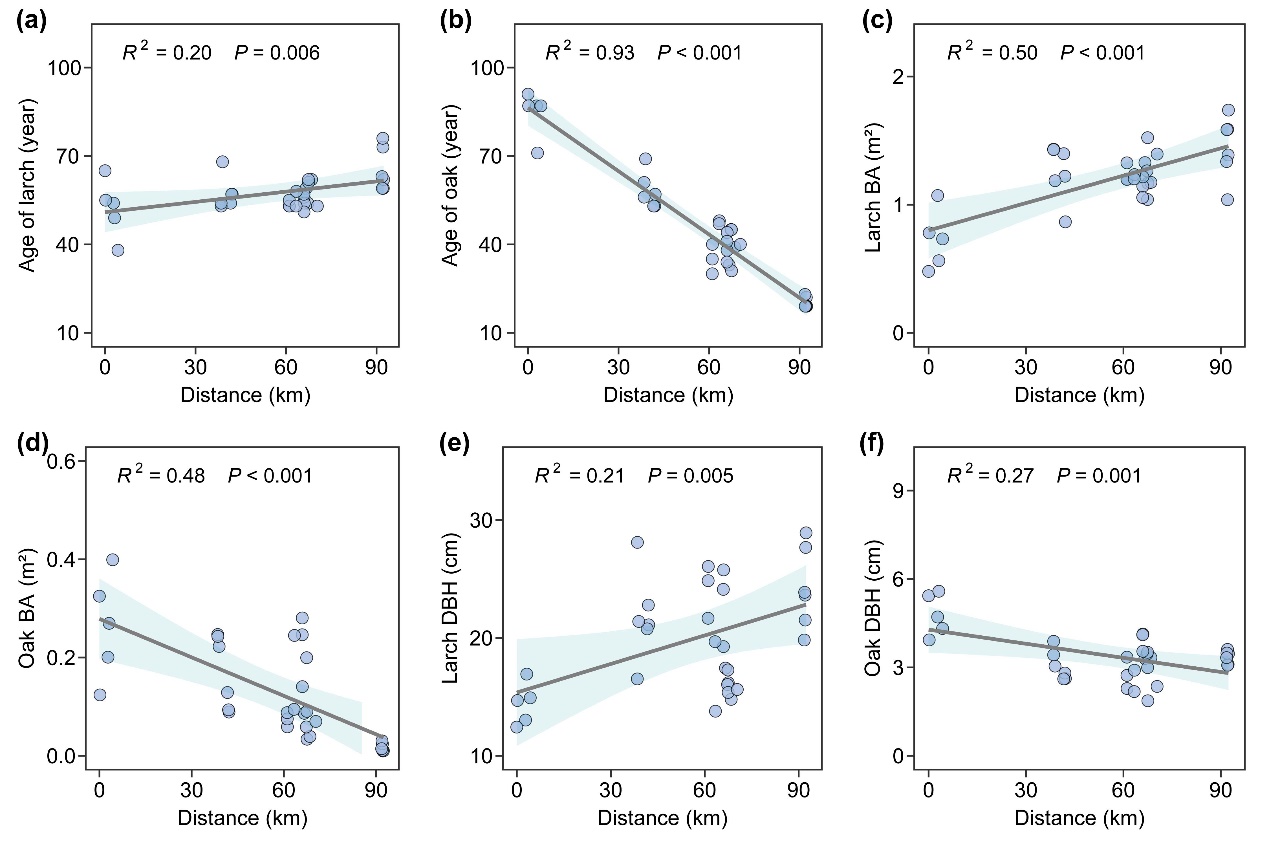


**Figure S3. Linear regression plots for (a) MAT and (b) MAP between observed meteorological data and CHELSA dataset for Area 1 (i.e. Jagdaqi).**


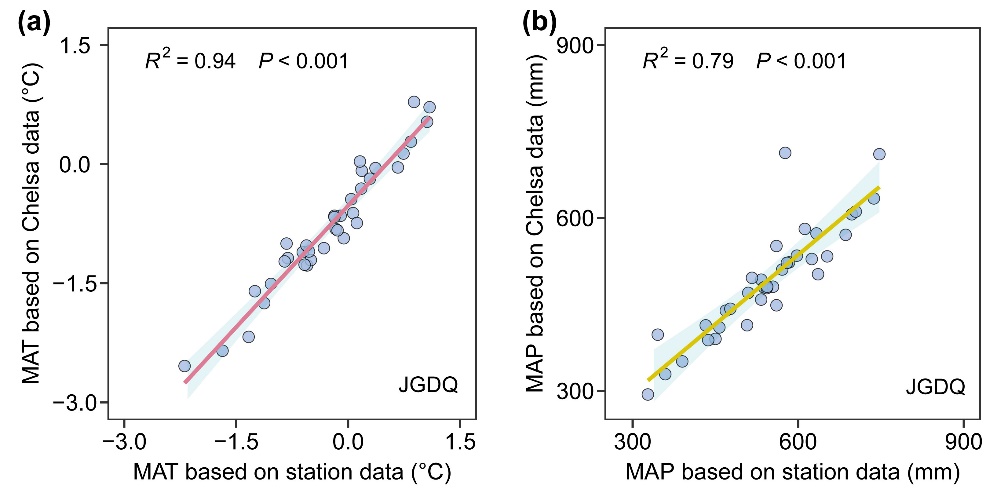


**Figure S4**. **Pearson correlation coefficient of driving factors.** See the caption of Figure S1 for abbreviations.


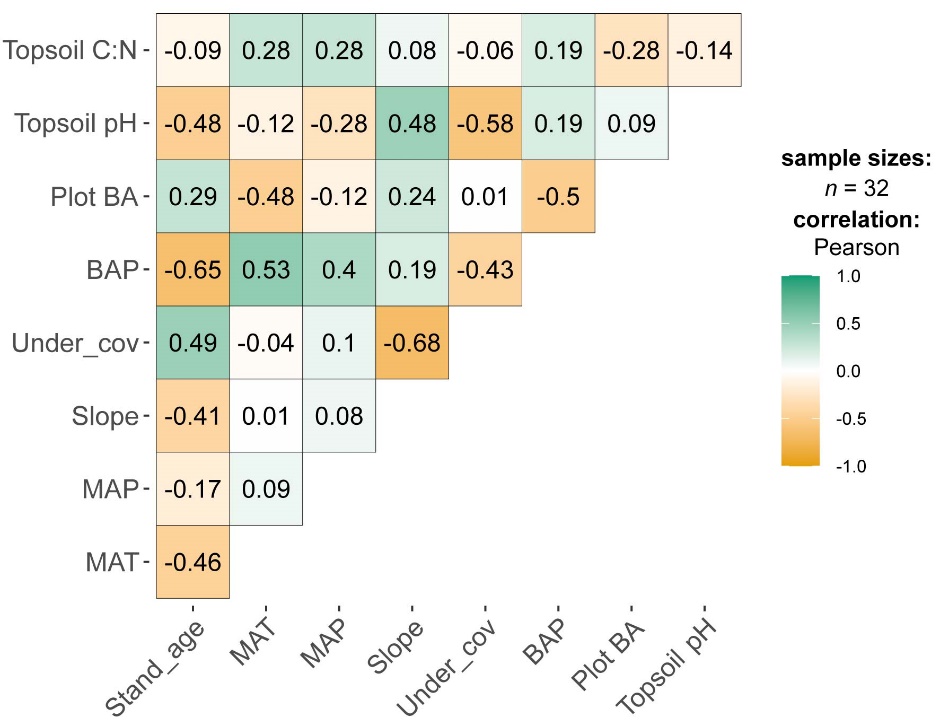


**Figure S5. Frequency distribution of topsoil C:N ratio and** δ**^15^N across the sampling transect.**


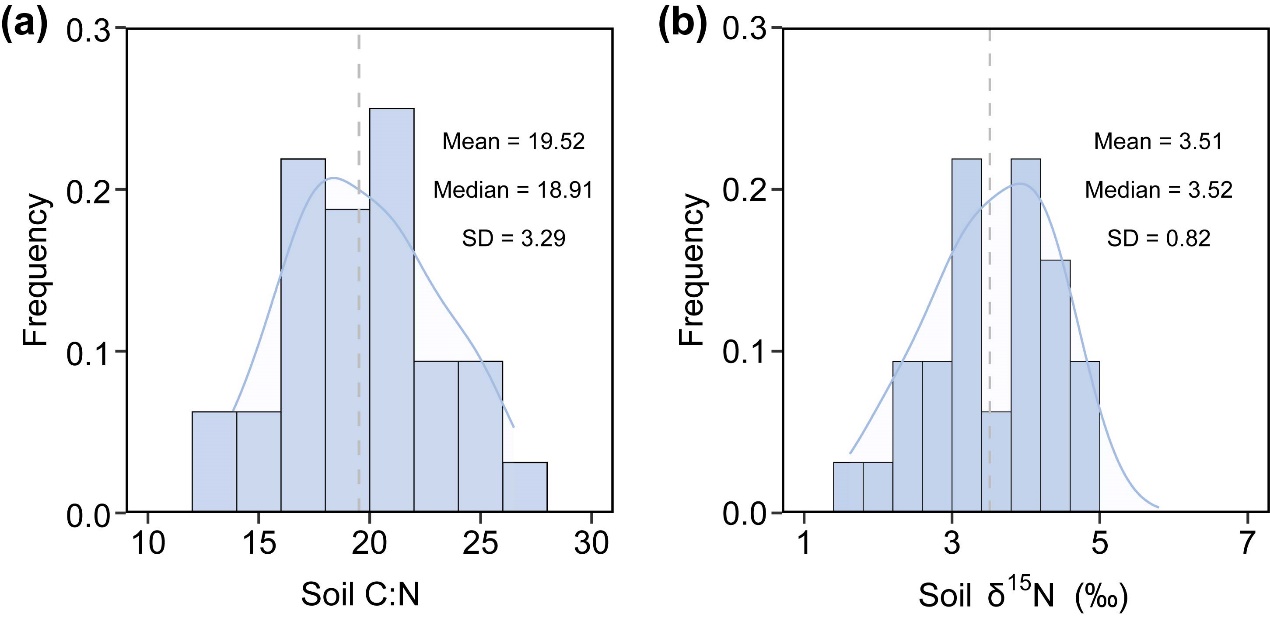


**Figure S6. Frequency distribution of foliar C:N ratio for Mongolian oak and Dahurian larch across the sampling transect.**


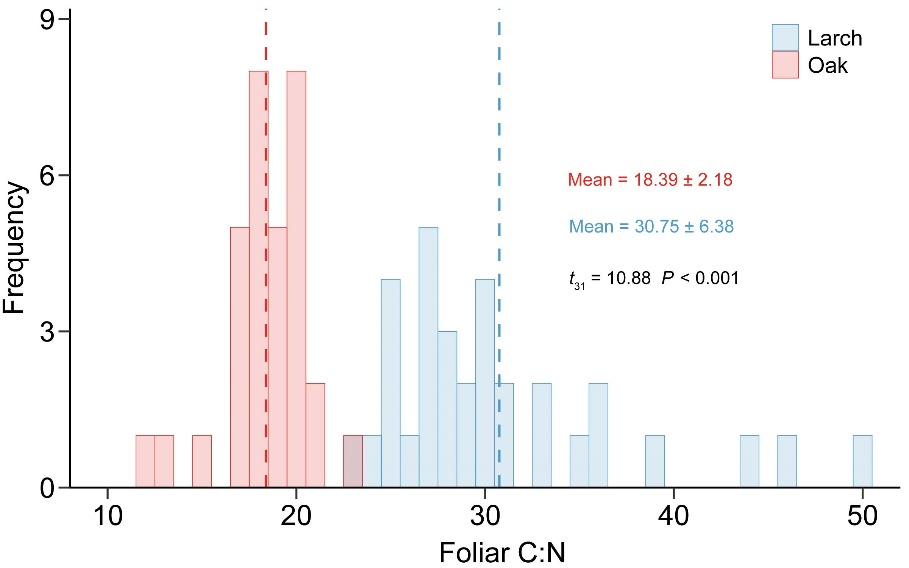

Supplement: Supplementary file 1 [file mmc1.docx]
